# Madrid International Plan of Action on Ageing and the 2030 Agenda for Sustainable Development

**DOI:** 10.3389/ijph.2024.1607078

**Published:** 2024-10-10

**Authors:** Karoline Schmid

**Affiliations:** United Nations, New York, NY, United States

**Keywords:** population ageing, sustainable development, Africa, Madrid International Plan of Action on Ageing, United Nations

## Demographic Trends

Population aging is one of the most significant global trends of our era. People are living longer and reaching older ages than ever before. This shift reflects our remarkable collective achievements in enhancing living conditions for billions worldwide. Improvements in sanitation, medical treatments, access to education, family planning, and progress in gender equality and women’s empowerment have all played crucial roles in the transition from high to low fertility and mortality rates. These advancements have marked the beginning of an era where rapid population growth is gradually slowing, leading to a steady increase in the proportion of older individuals in society [1].

## Madrid Plan of Action and the 2030 Agenda for Sustainable Development

The United Nations has a long history of recognizing the importance of population ageing for development. The First World Assembly on Ageing convened 40 years ago in Vienna, Austria was the first global effort to comprehensively address the issues of ageing. It recognized ageing as a global phenomenon and outlined actions to improve the wellbeing and quality of life of older persons and aimed at providing opportunities for older persons to contribute to national development. The Vienna Plan of Action was the result of the World Assembly and was also endorsed by the United Nations General Assembly in 1982 [2].

The Second World Assembly on Ageing, held in Madrid, Spain in 2002, reiterated many principles from the Vienna Plan, including dignity, independence, participation, care and the elimination of discrimination and highlighted intergenerational solidarity. The Assembly concluded with the adoption of a Political Declaration and the Madrid International Plan of Action on Ageing [3] that contains an International Strategy for Action on Ageing. MIPAA provides practical assistance to policymakers in dealing with the demographic shifts in their societies and calls on all stakeholders to mainstream ageing into all national policies and development programs. The MIPAA spans a broad range of issues relevant for ageing societies, emphasizing three priority areas: older persons and development; advancing health and wellbeing into old age; and ensuring enabling and supportive environments. A critical principle to the MIPAA strategy is the bottom-up approach that involves implementing MIPAA at the local and national levels, taking local context into consideration when developing policies and programs. It also requires the active involvement and participation of various stakeholders, including older persons themselves, working on customized solutions that reflect local knowledge, customs and traditions. Of critical importance is continued monitoring and evaluation to help identify what is working, what needs adjustment and what lessons can be shared with others.

The Commission for Social Development of the United Nations Secretariat is tasked to undertake every 5 years the MIPAA review and appraisal at the global level. Governments, the United Nations system and civil society participate in a bottom-up approach to assess progress made and challenges faced in the implementation of this plan. The most recent, the fourth review and appraisal, was undertaken in 2022 in observation of the 20th anniversary of the adoption of MIPAA. This review took place against the backdrop of a devastating coronavirus disease pandemic which directly or indirectly cost the lives of over 12 million older persons in 24 months, more than half of them in lower-middle-income countries [4]. One of the most salient conclusions from the fourth review and appraisal is that great disparities exist among and within regions in the rate of implementation of the MIPAA, in the focus of countries and regions around the issue of population ageing and on what constitutes an emerging issue or an ongoing challenge in each context. The assessment further found that inadequate national institutions and institutional mechanisms as well as age-based discrimination remain a concern. Important for effective and efficient policy making and implementation are a strong knowledge base and high-quality age-disaggregated data.

The most recent review undertaken by the United Nations Economic Commission for Africa (ECA) emphasized that while Africa is the youngest continent, its older population is growing rapidly and cautioned that the gains in economic growth could be lost if Africa did not plan for its growing older populations. The review further found that only about one-third of African countries had strategies to implement the targets set in MIPAA, that these strategies needed to be aligned with the legal national frameworks and that international aid could boost the impact of government initiatives in support of older persons. The review also showed that even after 20 years since the adoption of MIPAA, older Africans are typically not supported by social and development programs. Also, the region is not adequately investing in efforts to support the rights and needs of its older population. The continent still needs to recognize that while older people have challenges, they also contribute to the continent’s development. Findings further highlight that the COVID-19 pandemic has often left older people in many African countries without income, food and other forms of support [5].

As the United Nations are accelerating their efforts towards achieving the pledge to leave no one behind made when the international community adopted the 2030 Sustainable Development Agenda [6] and the related Sustainable Development Goals in 2015, the Madrid Plan of Action at 20 continues to offer a solid foundation to entice Governments to bring about the far-reaching social, economic, environmental and political changes needed to fully capture the realities of population ageing. While progress has been made towards implementing MIPAA and achieving the 2030 Sustainable Development Agenda, Governments, civil society and academia need to collaborate to take advantage of these demographic changes while at the same time preparing for the challenges population ageing poses.

The role of academia is critical for providing in-depth and inter-disciplinary research to enhance the knowledge and understanding of population ageing. Investigating and tapping into local communities that often possess valuable knowledge about the needs, preferences, and challenges of their older residents will benefit the development of better informed and more effective policies and interventions. Policies implemented in response to this historic global trend can be harnessed to uphold the pledge contained in the 2030 Agenda for Sustainable Development that no one will be left behind. Now is the time to plan for the long-term, to prepare for the challenges ahead and to take advantage of the opportunities presented.
